# Indigenous women's mental health across the life course: a global policy brief for rights-based, culturally safe care

**DOI:** 10.3389/fgwh.2025.1691146

**Published:** 2026-01-14

**Authors:** Miranda Field

**Affiliations:** Educational Psychology, University of Regina, Regina, SK, Canada

**Keywords:** culturally safe care, indigenous, indigenous data sovereignty, life course approach, mental health, women health

## Abstract

Indigenous women experience distinctive mental health risks that accumulate across the life course under the continuing impacts of colonization, gendered violence, and systemic racism. Drawing on recent mandates from the United Nations Permanent Forum on Indigenous Issues and the World Health Assembly's Resolution 76.16 (2023), as well as community-based exemplars such as Partners In Health's women-led peer models, this policy brief applies the analytical dimensions of the National Collaborating Centre for Healthy Public Policy to synthesize evidence, contextual factors, and feasible policy options. It identifies disproportionate burdens in suicide rates, perinatal depression, caregiver stress, and menopausal symptom severity, alongside a persistent lack of validated Indigenous-specific screening tools and gender-disaggregated data. The brief recommends an integrated, rights-based strategy that funds Indigenous governance of culturally safe mental health services across the life course, builds an Indigenous Women's Mental Health Data Strategy grounded in data sovereignty, embeds traditional knowledge and place-anchored healing in coverage policies, and extends targeted support for caregiving and menopausal transitions. Implementing these measures would operationalize reconciliation commitments, reduce documented inequities, and generate long-term social and economic benefits for communities and health systems alike.

## Introduction

Globally, Indigenous women face disproportionate mental health burdens across the life course, yet international, national, and local systems rarely provide culturally safe, community-led, and gender-responsive care. Therefore, action that centers around decolonization, reconciliation, and strength-based Indigenous models is urgently required. Without targeted, Indigenous-led action, preventable mental health morbidity and avoidable deaths among Indigenous women will continue to rise within the next decade, despite existing global mandates.

This brief translates global mandates and available evidence into actionable policy options for Indigenous women's mental health across the life course. It is written for policymakers, Indigenous governing bodies, health system leaders, researchers, and students in diverse regions. It provides a structured, decolonizing pathway to design, fund, and evaluate culturally safe, community-led, gender-responsive care by tailoring it with local supporting information.

## Background

### Global mandates

The United Nations (UN) Permanent Forum on Indigenous Issues has recognized “the urgent need to increase commitment to the health of Indigenous women globally” and urged Member States and UN entities to finance culturally appropriate services and midwifery programs (1). In 2023, the World Health Assembly (WHA) passed Resolution 76.16, directing the World Health Organization (WHO) to draft a Global Plan of Action for the Health of Indigenous Peoples using a life course approach, with special attention to reproductive, maternal, and adolescent health (2).

### Rights framework

The UN Declaration on the Rights of Indigenous Peoples (UNDRIP) affirms the right to the “highest attainable standard of physical and mental health” and to maintain traditional health practices.

### Indigenous wellbeing

Indigenous wellbeing refers to Indigenous-led health practices rooted in relationships with land, waters, and place. The term “place-anchored healing,” used in this brief, encompasses ceremony, language, kinship, and stewardship, and is implemented with the consent and governance of the Indigenous Peoples of that Place.

### Implementation exemplars

Partners In Health (PIH) delivers community-based, rights-based mental healthcare in 11 countries, integrating traditional healers, task-sharing groups, and women-led peer groups (2022). Waminda (Aboriginal Women's Health and Wellbeing) provides an Indigenous community–-controlled Birthing on Country redesign grounded in trauma- and violence-informed decolonizing practice, demonstrating feasibility and cultural safety within a place-anchored model (3–5). Nato’ we ho win (The Art of Self-Healing, Canada) demonstrates that a culturally anchored intervention can measurably improve mental health outcomes for Indigenous women who have experienced intimate partner violence (IPV), with effects observed up to 1 year later (6).

## Research approach

This brief views Indigenous women's mental health through a life course, decolonizing lens that positions colonization, gendered violence, and systemic racism as structural determinants of health. Evidence is drawn from peer-reviewed and authoritative gray literature, prioritizing Indigenous-led or codesigned studies and policy exemplars. Then, this evidence is synthesized across four streams: (1) international mandates and guidance (e.g., UNDRIP and WHA 76.16), (2) peer-reviewed literature, (3) Indigenous- and community-generated knowledge, and (4) policy/practice exemplars.

Analytically, we map outcomes across the life course (childhood, adolescence, perinatal period, caregiving, perimenopause/menopause, and elderhood) to identify burdens, gaps, and leverage points. We then apply the National Collaborating Centre for Healthy Public Policy (NCCHPP) dimensions (effectiveness, equity, costs, feasibility, acceptability, and unintended effects) to compare options. Next, we center Indigenous governance, treating Free, Prior, and Informed Consent (FPIC) and Indigenous data sovereignty as non-negotiable implementation criteria. Finally, we consider intersectionality (e.g., rurality/urbanization, socioeconomic status, and Two-Spirit/LGBTQIA+ identities) where data permit.

## Research findings

To advance our goal of translating global mandates and evidence into action, this section synthesizes life course evidence on Indigenous women's mental health, highlighting where burdens are concentrated and where data gaps limit decision-making.

Table 1 presents life course stages and transitions and highlights key mental health challenges likely encountered by Indigenous women at each stage. It also provides examples of literature and programs for each stage.

**Table 1:** Life-course stages and transitions—key mental-health challenges for Indigenous women.

Stage/transition	Key mental health challenges	Illustrative data
Childhood and adolescence	Historical trauma, discrimination, and suicide risk	Indigenous youth suicide rates are above national averages in several Organisation for Economic Co-operation and Development (OECD) countries (7, 8).
Young adulthood	Urban migration stress and gender-based violence	Intimate partner violence is a leading risk factor for PTSD, depression, and suicide among American Indian/Alaska Native women (9, 10).
Motherhood and perinatal period	Perinatal depression/anxiety and maternal mortality	Indigenous women experience postpartum depression 87% more often than white women, with maternal deaths frequently linked to mental health causes (11–13). Birthing on Country models demonstrate improved outcomes relative to standard care (e.g., reduced preterm birth and higher breastfeeding) (14–16). Community-controlled exemplars such as Waminda illustrate culturally safe implementation pathways (4).
Caregiving	Dual caregiver stress and economic insecurity	Caregivers report higher frequent mental distress and diagnosed depression than non-caregivers [(17), August 29].
Perimenopause and menopause	Underexplored emotional fluctuations, stigma, and symptom severity	Indigenous women report menopause symptoms as severe or higher than other racialized groups (18, 19) and seek culturally congruent remedies (20, 21).
Elder years	Isolation and chronic disease comorbidity	WHO notes a 20-year life expectancy gap and high disability rates among older Indigenous people (2023).

## Key considerations

### Research (effectiveness, equity, unintended effects, and evidence gaps)

Consistent findings show a higher prevalence of depression, anxiety, and post-traumatic stress disorder (PTSD) among Indigenous women, amplified during reproductive and menopausal transitions (22, 23). Indigenous-specific screening tools remain scarce (24). Evidence on menopause-related mental health for many Indigenous Peoples remains limited, underscoring the need for gathering local data. Codesigned perinatal social and emotional wellbeing (SEWB) screening approaches such as the Baby Coming You Ready (BCYR) digital platform illustrate strengths-based, context-specific assessment under Indigenous governance. Impact evidence is emerging and should be locally evaluated (25, 26).

### Organizational capacity (effectiveness, costs, feasibility)

Community-run services (e.g., PIH women's circles) demonstrate feasibility and cost-effectiveness by leveraging Indigenous workforce and task-sharing models (27).

### Political landscape *(feasibility)*

United Nations Permanent Forum on Indigenous Issues (UNPFII), WHO, and several Member States endorse Indigenous leadership, yet funding flows and disaggregated data remain limited (1, 2).

### Community values *(acceptability)*

Strength, relationality, and place-anchored wellness are central. Interventions that privilege these values achieve higher uptake and satisfaction (28).

Building on these considerations, we broadly apply the NCCHPP framework to evaluate policy options—assessing dimensions of effectiveness, equity, costs, feasibility, acceptability, and unintended effects to guide actionable choices (29). Table 2 offers evidence-informed examples for each NCCHPP dimension. These are illustrative judgments drawn from the literature and practice and not a full systematic appraisal. These dimensions can guide local adaptation and deeper assessment where warranted.

**Table 2:** NCCHPP policy-analysis dimensions—summary of evidence assessment.

Dimension	Assessment
Effectiveness	Evidence supports community-led, culturally adapted models for maternal, youth, and menopause care (15, 23).
Equity	Current systems widen gender–ethnic gaps; Indigenous models reduce inequities by 20%–40% in pilot sites (15, 16).
Costs	Community programs such as Nato’ we ho win report low start-up costs (facilitator training, elder honoraria, cultural materials, and space), with ongoing expenses per cohort driven mainly by staffing, child care, and transportation. As a low-capital, community-delivered intervention, its costs are modest compared with the high downstream costs of untreated IPV-related trauma and mental illness (6).
Feasibility	Legal mandates exist (UNDRIP and WHA 76.16). Workforce can be scaled through task-sharing and blended training (30–32). Codesigned, digital SEWB screening platforms (e.g., BCYR) indicate feasible integration when governed by Indigenous organizations and aligned with data sovereignty protocols (25, 26).
Acceptability	Acceptability is high among Indigenous communities; there is rising public and governmental support for reconciliation commitments [(33, 34), Nov 4]. Community-controlled models (e.g., Waminda) report high engagement and cultural safety, supporting acceptability (4).
Unintended effects	There is a risk of cultural appropriation if Indigenous governance is weak, which underscores the need for Free, Prior, And Informed Consent (FPIC) and adherence to Indigenous data sovereignty principles (35), as emphasized by the United Nations Educational, Scientific And Cultural Organization (36).

## Recommendations

Translating analysis into action, this brief presents prioritized, rights-based policy options that can be adapted locally under Indigenous governance (Table 3).

**Table 3:** Prioritized rights-based policy options adaptable under Indigenous governance.

Option	Pros	Cons/risks	NCCHPP lens
1. Invest in community-led, culturally safe mental health services across the life course (e.g., Indigenous midwifery, youth circles, and elder support)	High effectiveness; aligns with rights; builds local capacity	Sustained funding and Indigenous governance structures required to offset risks	Effectiveness, equity, and acceptability
2. Establish a national Indigenous Women's Mental Health Data Strategy (ethnicity- and gender-disaggregated indicators and Indigenous data sovereignty protocols)	Enables accountability; fills data gaps	Fear of data misuse and tech infrastructure	Feasibility, equity, and unintended effects
3. Integrate traditional knowledge and place-anchored healing into health system policies (see TRC Call to Action #22 call-out) (coverage for midwives, traditional medicines, ceremony)	Advances reconciliation; improves engagement	Regulatory adjustments needed; provider training gaps	Acceptability and feasibility
4. Expand caregiver and menopause supports (peer groups, flexible benefits, culturally tailored education)	Addresses neglected life course stages; leverages existing community networks	Limited models to scale; cross-sector funding may be required to offset this disadvantage	Equity and cost-effectiveness

“We call upon those who can effect change within the Canadian health-care system to recognize the value of Aboriginal healing practices and use them in the treatment of Aboriginal patients in collaboration with Aboriginal healers and Elders, where requested by Aboriginal patients (37).”

## Recommended action

Adopt Option 1 as the cornerstone, supported by Options 2–4 in a phased, rights-based implementation plan. This integrated approach includes the following:

1. funds Indigenous governance to design, deliver, and evaluate services;
2. implements WHA 76.16 through a life course model that embeds traditional knowledge and strength-based practices;
3. builds robust data systems to monitor outcomes while upholding Indigenous data sovereignty principles; and
4. addresses underserved transitions (perinatal, caregiving, and menopause) through tailored support.

Federal/national, provincial/territorial, and local governments should codevelop agreements with Indigenous women's organizations, leveraging PIH, WHO, and UN resources for support. Immediate priorities include allocating targeted funds in the next budget cycle, instituting Indigenous leadership seats on mental health governance bodies, and embedding cultural safety competencies in all relevant workforce standards.

## Implementation and adaptation

This section bridges global principles with locally implementable action. Building on the brief's aim to translate global mandates and evidence into actionable, life course policy, this section guides readers in adapting the aforementioned recommendations to their legal, cultural, and health system contexts under Indigenous leadership. This brief synthesizes diverse sources across regions; however, data remain uneven and Indigenous-specific screening tools are scarce. Evidence on menopause-related mental health is limited for many Indigenous Peoples. Findings emphasize transferability principles rather than uniform effect sizes; local codesign, FPIC, and data governance are essential to contextualize impacts. Adaptation should be anchored in WHA 76.16's life course emphasis and the forthcoming WHO Global Plan of Action on the Health of Indigenous Peoples to provide a shared cross-regional mandate that supports transferring the recommendations across jurisdictions under Indigenous leadership. What follows must be used to align with international obligations (e.g., UNDRIP and WHA 76.16), FPIC and data sovereignty must be upheld, and service design, workforce approaches, and coverage policies must be tailored that so they are place-anchored, feasible, and equitable. Where impact evidence is emerging (e.g., perinatal SEWB digital screening), it must be piloted under Indigenous governance with FPIC and evaluated using gender- and identity-disaggregated indicators.

1. Map mandates (UNDRIP; WHA 76.16) to local law.
2. Confirm Indigenous governance and FPIC.
3. Select Options 1–4 based on local capacity and codesign.
4. Consider adaptations such as governance models and Indigenous leadership; recognition/legal status of traditional healers; workforce and task-sharing pathways; financing/benefit coverage; data sovereignty and privacy; terminology/language use; referral pathways and service integration; regulatory scope and credentialing; training/supervision requirements; urban–rural/remoteness factors; and monitoring indicators for equity and effectiveness.

## Conclusion

This brief integrates global mandates, community evidence, and a decolonizing life course analysis to translate principles into practical, locally adaptable actions under Indigenous leadership. Implemented as recommended—through funding Indigenous governance, embedding traditional knowledge and data sovereignty, and targeting perinatal, caregiving, and menopausal transitions—these options can operationalize reconciliation commitments, reduce documented inequities, and generate long-term social and economic benefits for communities and health systems alike, while strengthening culturally safe care across jurisdictions**.**

Once customized, this brief can serve multiple purposes: It can be submitted to officials (e.g., Ministers of Health, Indigenous affairs offices, federal/provincial/state/territorial departments, professional colleges, and multilateral agencies) as a concise, evidence-based call to action; used as a teaching case in public health, policy, or Indigenous studies courses to practice contextual adaptation and stakeholder analysis; and shared with community organizations to support grant applications, strategic-planning sessions, and codeveloped advocacy campaigns.

Taken together, these pathways represent the immediate route from shared global commitments to place-anchored implementation. Action taken now can stem preventable morbidity and avoidable deaths, while improving Indigenous women's mental health across the life course.

### Preamble: how to use and adapt this policy brief

This policy brief is intentionally written for inclusion in an international, multidisciplinary issue on women's health. Its purpose is twofold:

1. A model for action-oriented scholarship

By integrating global human rights mandates (e.g., UNDRIP and WHA 76.16) with community-generated evidence and a decolonizing, strength-based lens, the brief demonstrates how scholarship can move beyond description toward actionable policy recommendations. Readers can use its structure—issue framing, life course analysis, NCCHPP (Canada) policy dimensions, and an options matrix—as a template when tackling other complex health equity challenges.

2. A ready-to-tailor advocacy tool

Although the data and exemplars cited span multiple regions, every section is designed for easy localization. Users are encouraged to do the following:

• Insert local indicators (e.g., Indigenous nation population size, suicide rates, and maternal mortality figures) to create jurisdiction-specific urgency;
• Replace or augment case studies with community-led initiatives from their own context;
• Align terminology with national legal frameworks and preferred Indigenous language(s); and
• Map recommendations onto existing budget cycles, strategic plans, or reconciliation commitments.
